# Breeding and Ontogeny of the Aquarium-Traded Scissortail Rasbora (*Rasbora trilineata*)

**DOI:** 10.3390/ani15131823

**Published:** 2025-06-20

**Authors:** Krittima Kasamawut, Suriya Udduang, Supalug Kattakdad, Kasama Danwandee, Achara Jutagate, Samnao Saowakoon, Tuantong Jutagate

**Affiliations:** 1 Faculty of Agriculture and Technology, Rajamangala University of Technology Isan Surin Campus, Muang, Surin 32000, Thailand; krittima.sa@rmuti.ac.th (K.K.); suriya.ud@rmuti.ac.th (S.U.); supalug.ka@rmuti.ac.th (S.K.); kasama.da@rmuti.ac.th (K.D.); 2 Faculty of Agriculture, Ubon Ratchathani University, Warin Chamrab, Ubon Ratchathani 34190, Thailand; achara.j@ubu.ac.th

**Keywords:** *Rasbora trilineata*, synthetic hormone Suprefact^®^, embryonic development, larval development

## Abstract

Breeding and ontogeny development of the scissortail rasbora (*Rasbora trilineata*) was studied for conservation and enhancement purposes. A wild broodstock was collected from a reservoir in Surin Province, Thailand. Breeding was done by injecting the synthetic hormone Suprefact^®^ to induce spawning. The suitable dosages for mature females and males were 15 and 7.5 µg per kg of fish, respectively (*p* < 0.05), compared to other treatments measuring numbers of spawned eggs, fertilization rates, and survival rates at 24 h. Chlorella was used to nurse the larvae for 14 days after hatching (DAH). Moina was used to rear the larvae until they reached 40 days old since this provides significantly higher (*p* < 0.05) survival rates comparable to other artificial feeds in the experiment. For ontogeny, this study documented cleavage, blastula, gastrulation, segmentation, and the pharyngula period, which culminated in hatching within 18 h at 28–30 °C. The larvae exhibited rapid growth, reaching 2 cm in total length by 40 DAH, which is suitable for trading or stocking for resource enhancement.

## Simple Summary

Small-sized fish with a maximum length of around 10 cm are native to Southeast Asia and are popular as ornamental fish. Due to the decline of the wild populations, this study investigated a method for artificial breeding, stimulated using the synthetic hormone Suprefact^®^, with dosages of 15 and 7.5 μg per kg for mature females and males, respectively. The green algae “Chlorella” was used as their feed for the first 14 days after hatching, followed by the small crustacean, “Moina”, until they were 40 days old, with a juvenile size of around 2 cm. Moreover, this study also documented the key developmental stages from fertilization to hatching as well as development from the larval to juvenile stages. These findings provide essential knowledge for developing breeding programs and reducing dependence on wild populations for the aquarium trade.

## 1. Introduction

Fishes of the genus *Rasbora* in the Cyprinidae family comprise 88 species. They are small-sized cyprinids. Their adult size is commonly around 10 cm in total length (TL) [1]. *Rasbora* is a highly speciose genus, with many species exhibiting similar morphological characteristics, often leading to taxonomic confusion [2,3]. Nevertheless, scissortail Rasbora (*Rasbora trilineata*) has distinct characteristics and is among the common Rasborin cyprinids. *R. trilineata* is a small freshwater fish native to Southeast Asia. The adults are typically between 5 and 10.5 cm in TL and can reach a maximum of 13 cm in TL [1]. This fish species is characterized by a dark dorsal coloration, silvery scales, and a distinctive black longitudinal stripe along its body [4]. *R. trilineata* is globally distributed due to its popularity as an ornamental fish, owing to its striking appearance, non-aggressive nature, and high adaptability under aquarium conditions [1]. This fish species exhibits a peaceful schooling behavior. It is commonly found in streams and waterfall pools and actively feeds near the water surface [5]. In nature, maturation of *R. trilineata* peaks once a year, from May to August [6]. Moreover, *R. trilineata* is closely related to the zebrafish *Danio rerio*, which suggests its potential for use as a fish model in biomedical research [7]. The chromosomes, phylogeny, mitochondrial genome, and DNA code of *R. trilineata* have already been reported, highlighting its feasibility for use as an aquatic animal model [2,3,8,9].

Owing to its attractive characteristics, which make it highly sought-after in the ornamental fish trade, wild populations of *R. trilineata* have shown a significant decline [2,3,6]. Moreover, many anthropogenic stressors, especially urbanization and climate change, result in the deterioration of their habitats [6]. These factors highlight the need for conservation strategies and integrated information on spawning and life history for resource enhancement. The protocols for resource enhancement of inland water bodies in Asia have been reviewed and presented. Environmental engineering and fish stocking programs are two main techniques for stock enhancement of inland fisheries [10]. For the latter technique, a sustainable breeding program and an understanding of larval development are required to guarantee the seed production and survival of the hatchlings before their release into natural waterbodies [11].

Breeding techniques for Rasborin cyprinids in hatchery environments are varied. They include adding another fish species as a stimulus [12], imitating preferred natural conditions in a breeding tank [13], mixing hormones with feed [14], injecting hormones [15,16], and hormone immersion [17]. Nevertheless, these studies are not focused on *R. trilineata*. Several *Rasbora* spp., such as *Rasbora lateristriata* [18], *Rasbora daniconius* [19], and *Rasbora argyrotaenia* [20], were studied during their embryonic development, but not yet for *R. trilineata*. The current study examines artificial breeding through injection and the embryonic development of *R. trilineata*. This is done to support sustainable aquaculture and conservation efforts. These study findings will contribute to the long-term conservation of this species while promoting responsible fishery management.

## 2. Materials and Methods

### 2.1. Source of the Broodstock

**Figure 1:**
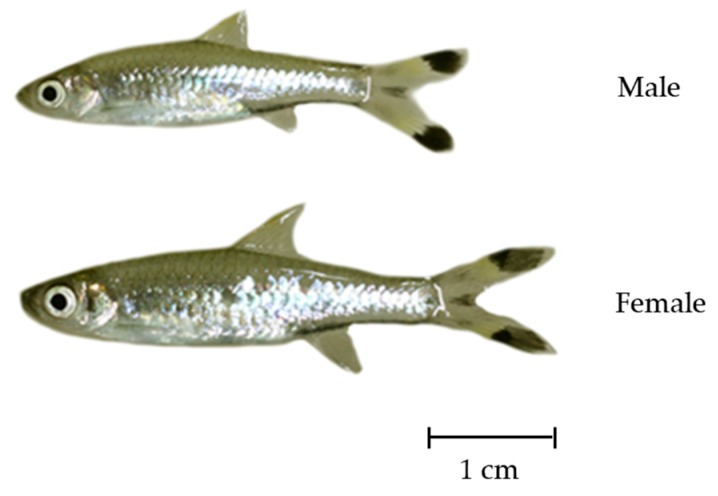
Mature male and female scissortail rasbora (*Rasbora trilineata*).

Four hundred and fifty (185 males and 265 females) *R. trilineata* were collected from Huay Saneng Reservoir in Surin Province, Thailand (Lat. 14°48′44.3″ N Long. 103°30′04.1″ E) from May to August 2021. Then, they were transported to the fish farm on the RMUTI-Surin Campus. They were acclimated and reared in an aerated controlled facility (a 1.5 × 0.5 × 1 m tank). The water used in the facility was clean, filtered, and sterilized using UV light. Fish were fed with live food and trained to consume commercial pellet feed to prepare them for breeding and further studies on larval development. Since *R. trilineata* has no distinct genital papillae, broodstocks were sex-segregated by external characteristics (Figure 1). Males are slender, smaller in size, and have more distinct patterns on their bodies than females, while females have deeper bodies. These sex differences become pronounced in nature during the breeding season.

### 2.2. Breeding and Larval-Rearing Experiments

The experiments employed four treatments, each with three replicates. Each replicate used two males and one female as broodstock. Females were selected based on their plump bodies. The average total length and weight of the males were 7.26 ± 0.21 cm and 1.53 ± 0.18 g, respectively. They were 7.98 ± 0.25 cm and 2.64 ± 0.27 g, respectively, for females. In this study, hormonal induction was carried out using a combination of Luteinizing Hormone Releasing Hormone analog (LHRHa), i.e., Suprefact^®^, and Domperidone, i.e., a Motilium solution. LHRHa, a synthetic analog of gonadotropin-releasing hormone, stimulates the secretion of luteinizing hormone and follicle-stimulating hormone, which are both essential for the ovulation process. Domperidone acts as a dopamine antagonist, reducing the inhibitory effect of dopamine on gonadotropin release, thereby enhancing the action of LHRHa. This combination protocol was selected instead of using a commercial product, such as Ovaprim, to ensure precise control over the hormone dosage, offering flexibility and experimental accuracy. All healthy samples were weighed to accurately estimate the required dose of the synthetic hormone Suprefact^®^ (Sanofi Aventis, Frankfurt, Germany) for injection to induce spawning. The Suprefact^®^ doses for females involved four treatments using 5, 10, 15, and 20 µg per kg of fish, which were in the range of the dosages used for other *Rasbora* spp. [6,12,13,14]. The dosage was half this concentration for the males at each treatment. A Motilium solution was also injected into the fish at 10 mg per kg of fish. The injection was delicately done intraperitoneally with a 1 mL syringe. After injection, each set of broodstock was moved to a 12 × 24 × 15-inch aquarium, allowing them to mate and spawn naturally. In the spawning aquarium, straw, ropes, and nets were placed for the fish to lay eggs, and shading was provided. The temperature was maintained between 25 and 30 °C with a pH level between 6.0 and 6.5, according to the ranges in the natural habitats of *R. trilineata* [3,4,5].

One-day-old *R. trilineata* larvae from the best breeding treatment tests were further used in a larval-rearing experiment. This experiment lasted for 40 days until the fish sizes reached 2–3 cm, which is the common size for aquarium trade export and the size for stocking natural water bodies. In this experiment, 100 larvae were reared in 12 × 24 × 15-inch aquaria in replicate treatments. Starting on Day 3 after hatching, the larvae were fed Chlorella ad libitum for 14 days or longer according to the designated treatments. Each of the four treatments was done with three replicates that included Moina, an artificial feed for fish larvae (Brand Higrade 9006T, 42% protein, Charoen Pokphand, Samut Sakhon, Thailand), an artificial feed for shrimp larvae (Brand K-GHOST, 45% protein, Pattern Pet, Nakhon Pathom, Thailand), and an artificial feed for aquarium fish (Brand PRiMA super premium, 50% protein, Pattern Pet, Nakhon Pathom, Thailand). The feed from Day 14 onward was supplied to each aquarium ad libitum until the end of the experiment. Uneaten feed was siphoned out daily. The water level was maintained at 15 cm. Water quality was monitored weekly. All parameters were in acceptable ranges for aquaculture (Table S1).

The data collected from the breeding experiment were the number of spawned eggs, fertilization rates, hatching rates, and survival rates 24 h after hatching, and were calculated as follows:

Fertilization rate (%) = (Number of fertilized eggs/Total number of eggs) × 100

Hatching rate (%) = (Number of eggs that hatched/Number of fertilized eggs) × 100

Survival rate (%) = (Number of larvae at 24 h/Number of larvae at hatched) × 100

### 2.3. Data Analyses

The monitored variables for the larval-rearing experiment were the survival rates at days 14 and 40. All estimates were presented as the mean ± SD. The mean of each variable was tested for significant differences using ANOVA. A post hoc Duncan’s multiple range test was conducted if a significant difference was found at α = 0.05. Statistical analysis was conducted using R version 4.3.1 [21].

### 2.4. Observation of Embryonic and Larval Development

Ten fertilized eggs were carefully collected from the breeding tank using a dropper and a siphoning method to observe their developmental stages. The live eggs were microscopically examined (Olympus CX41) using image processing software (Magnus MIPS, version 1.0.0.0). The developmental stages of the embryos were continuously observed until they began twisting movements and eventually hatched. Changes in their morphological features were photo-recorded using a digital camera (Samsung ST70, Thai Samsung Electronics, Bangkok, Thailand) mounted on the microscope. Egg images were captured before fertilization and at 1 h intervals thereafter. Larval development was also captured and sketched from days 2 to 40.

## 3. Results

### 3.1. Spawning Success and Nursing of Larvae

**Figure 2:**
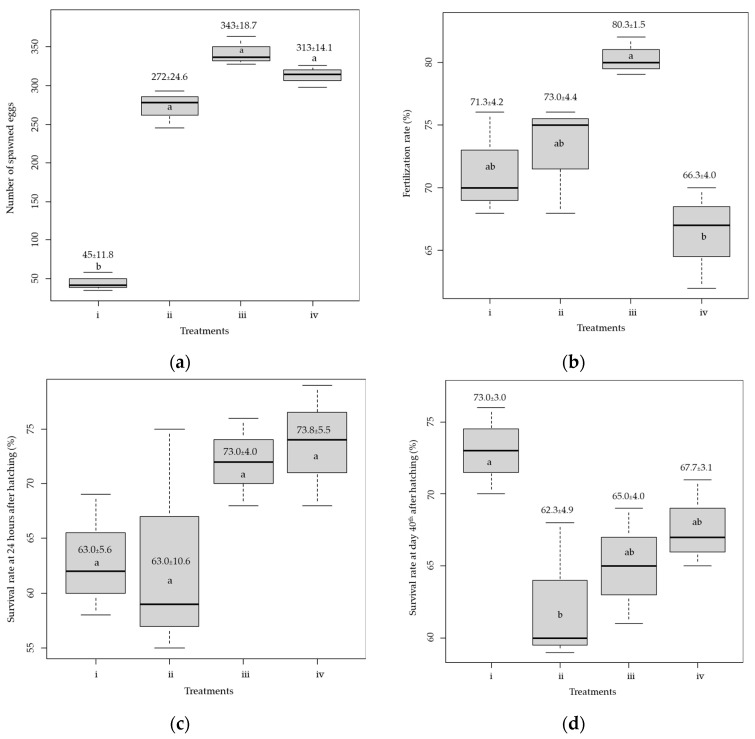
Graphical results from breeding and larval-rearing experiments: (**a**) number of spawned eggs, (**b**) fertilization rate, (**c**) survival rate at 24 h after hatching, and (**d**) survival rate at Day 40 after hatching. The dosages of Suprefact^®^ (µg per kg of fish) for females and males, respectively, in each treatment from Figure 2a to Figure 2c are (i) 5 and 2.5; (ii) 10 and 5; (iii) 15 and 7.5; and (iv) 20 and 10. Treatments for Figure 2d are (i) Moina, (ii) artificial feed for fish larvae, (iii) artificial feed for shrimp larvae, and (iv) artificial feed for aquarium fish. Numbers in each box plot indicate mean ± SD and the same letter of the box plots in each figure indicates a non-significant difference at α = 0.05.

The number of released eggs in the aquarium was counted. The number of spawned eggs from Treatment #1, i.e., 5 and 2.5 µg of Suprefact^®^ per kg, respectively, for females and males, revealed a significantly lower quantity (45.0 ± 11.8) than other treatments (*p* < 0.05, Figure 2a). The number of spawned eggs in the remaining treatments was not significantly different (*p* > 0.05), and the highest number of spawned eggs (343.0 ± 18.3) was found in Treatment #3, i.e., 15 and 7.5 µg of Suprefact^®^ per kg, respectively, for females and males. The fertilization rate was highest (80.3 ± 1.5%, Figure 2b) in Treatment #3 and lowest (66.3 ± 4.0%) in Treatment #4, i.e., 20 and 10 µg of Suprefact^®^ per kg, respectively, for females and males. The hatching rates were 100% across all treatments, but their survival rates varied. The survival rate was greatest for Treatment #4 (73.7 ± 5.5%), which was not significantly different from Treatment #3 (*p* = 0.53, Figure 2c). However, the survival for Treatments #1 and #2, i.e., 10 and 5 µg of Suprefact^®^ per kg, respectively, for females and males, was about 63.0%. Survival of the larvae during the first 14 days was over 85% for all treatments. However, nursing with Moina yielded the highest survival rate, 73.0 ± 3.0%, significantly higher than the other feeds (*p* < 0.05, Figure 2d).

### 3.2. Development of Eggs and Larvae

**Figure 3:**
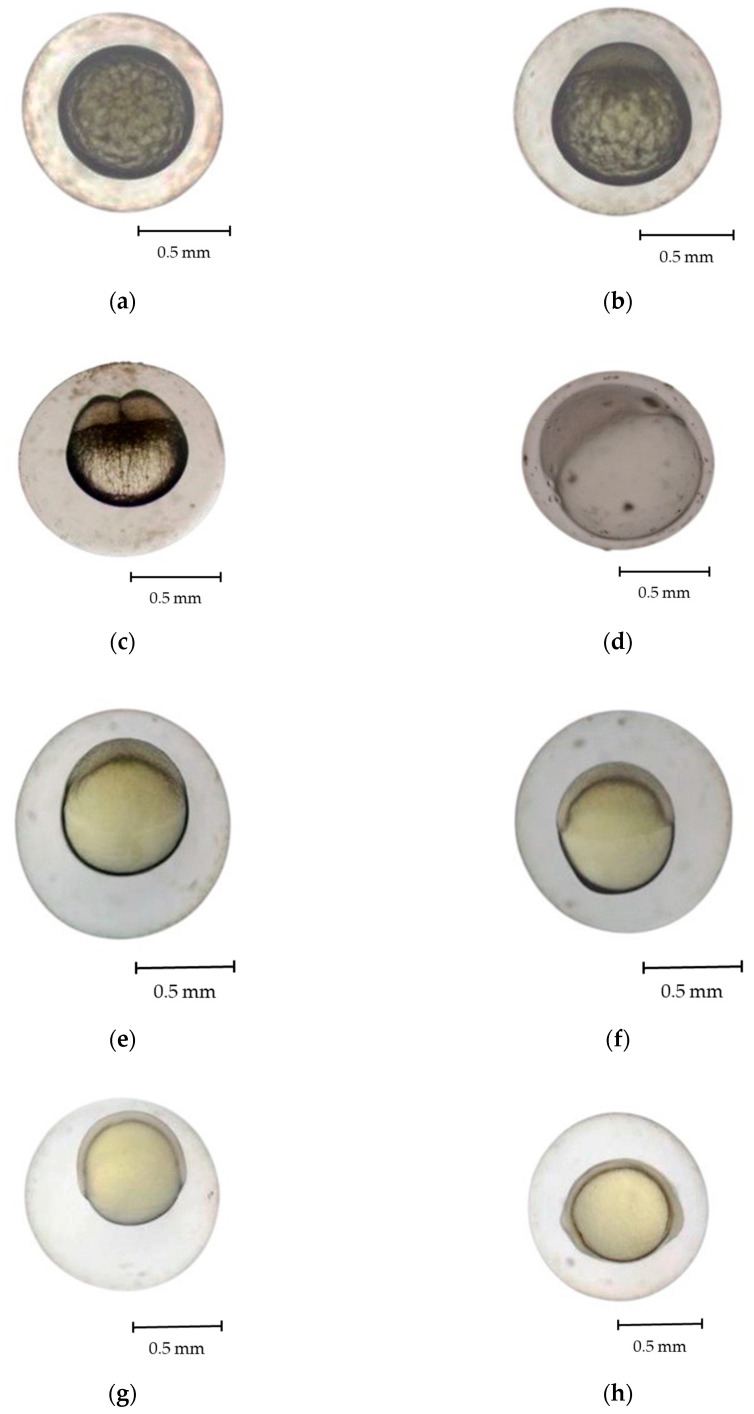

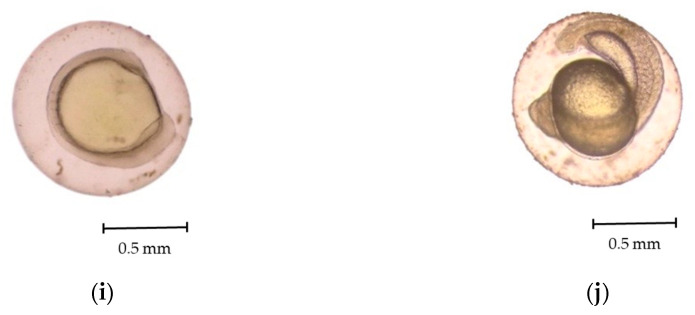
Embryonic development of scissortail rasbora (*Rasbora trilineata*), (**a**) fertilized egg, (**b**) zygote, (**c**) cleavage 2-cell period, (**d**) morula stage, (**e**) blastula period at 3.5 h, (**f**) dome stage at 4.5 h, (**g**) gastrula period cells organize into a germ layer, (**h**) gastrula period cells organize into a germ ring, (**i**) primordium at segmentation period, and (**j**) somite stage.

Fertilized *R. trilineata* eggs were thick, nonadhesive, spherically shaped, and transparent. They progressed through various developmental stages over approximately 18 h before hatching. The development stages of *R. trilineata* are zygote, cleavage, morula, blastula, gastrula, segmentation, pharyngula, and hatching. Fertilization of the eggs took place as soon as the sperm entered the eggs through the micropyle and when fusion between the two nuclei occurred. Almost immediately, the cortical reaction closed, and the micropyle denied entry to more sperm. Soon after fertilization (Figure 3a), the eggs swelled and started to develop. The key features at each stage are presented in Table 1 and described below.

- Zygote stage (Figure 3b)

This stage lasted from fertilization to the early cleavage stage, requiring approximately 10 min. Fertilization triggered cytoplasmic movements, causing the non-yolk cytoplasm to move toward the animal pole. The cytoplasm separated from the yolk at the vegetal pole, forming a structure called a blastodisc. The one-cell egg was visible from the dorsal view of the animal pole [22].

- Cleavage stage (Figure 3c)

Cleavage of eggs occurred within 0.5–2.0 h post-fertilization (hpf) at 28.0 °C. After cleavage, the eggs were meroblastic, forming a transitory blastula stage. Meroblastic cleavage occurs specifically in the blastodisc region of the embryo, with no cell division observable in the yolk, which is marked by a vertical cleavage extending from the animal pole toward the vegetal pole, terminating at the periphery of the yolk [18,23]. Vertical cleavage divided the blastodisc into two equal blastomeres within 2 hpf. During the cleavage period, sequential divisions occurred: the first at 13–15 min post-fertilization, producing two cells; the second at 17 min, forming four cells; and the third at 20 min, yielding eight cells. By 30 min, rapid divisions led to the morula stage.

- Morula stage (Figure 3d)

At this stage, the blastoderm began expanding as a thin sheet over the yolk surface. A distinct germinal ring developed, partially enclosing the yolk. The blastoderm eventually covered about half of it. A cap-like structure emerged at the animal pole and progressively enlarged. The embryo took on a mulberry-like appearance characteristic of the morula stage, which was observed at approximately 2.5 hpf.

- Blastula stage (Figure 3e,f)

During the blastula period, which occurred at 3.5 hpf, there was a noticeable increase in the number of cells as the cleavage process continued. As development progressed, the embryo entered the dome stage at 4.5 hpf. During this phase, the cells started organizing into a dome-shaped structure, indicating a shift toward a more orderly horizontal arrangement. Retnoaji et al. [18] reported that the epiblast and hypoblast layers are formed at this stage. The blastula period is essential for priming the embryo for subsequent developmental events, particularly the formation of germ layers during the gastrula stage.

- Gastrula stage (Figure 3g,h)

At approximately 5 hpf, the gastrula stage began, characterized by the formation of a germ ring as the cells organized into the primary germ layers. Also, in this stage, the blastomeres begin to migrate inward toward the yolk [18,23]. The gastrula phase is pivotal in establishing the embryo’s fundamental body plan, as cells begin differentiating into distinct layers that give rise to various tissues and organs. Kimmel et al. [24] reported that the embryonic shields, an accumulation of cells at one location within the germ ring, also occur during the gastrula period.

- Segmentation stage (Figure 3i,j)

Throughout the segmentation period, notable morphological changes occur as embryonic development progresses, marked by the formation of segmented structures known as somites. Various tissues, as well as vertebrae, skeletal muscles, and dermis, form during this stage [24]. At 8 h post-fertilization (hpf), the head bud stage begins with the formation of distinct head and tail regions. By 8.5 hpf, the primordium stage is marked by optic bud development. Somite formation initiates at 9 hpf along the embryonic axis and increases progressively, with a marked rise at 11.5 hpf. At 12 h 55 min, myotomes form, initiating muscle contractions. By 15.5 hpf, embryos exhibit spontaneous wriggling within the chorion, indicating early motor activity before hatching.

- Pharyngeal stage

This stage represents a phase where various organs form, both internal, e.g., vessels, pericardial cavity, and brain parts, and external, e.g., eyes, fins, skin, and pigments [18]. The pharyngula period of *R. trilineata* commenced at 17 hpf, during which the chorion membrane developed, offering structural protection to the embryo. By 18 hpf, hatching occurred, and the embryo emerged as a free-swimming larva, signifying the transition from the embryonic to the larval stage.

### 3.3. Larval Development

**Figure 4:**
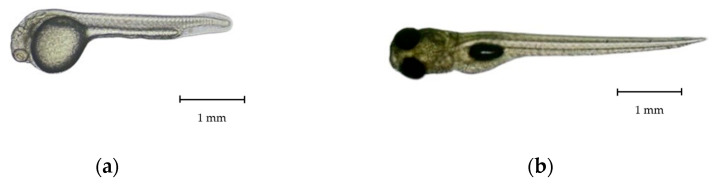
Larvae of scissortail rasbora (*Rasbora trilineata*): (**a**) 1 day old at a 3.84 ± 0.40 mm TL, and (**b**) 7 days old at a 6.13 ± 0.80 mm TL.

**Figure 5:**
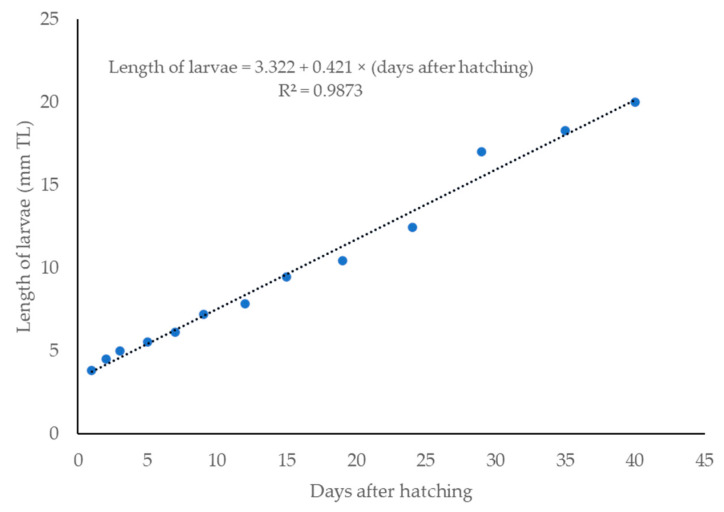
Lengths of scissortail rasbora (*Rasbora trilineata*) between days 1 to 40 after hatching.

**Figure 6:**
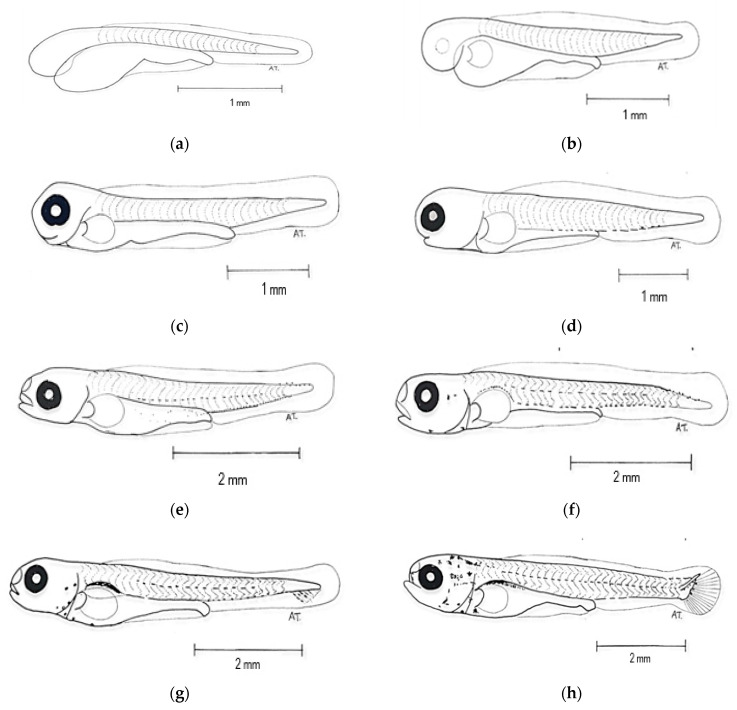

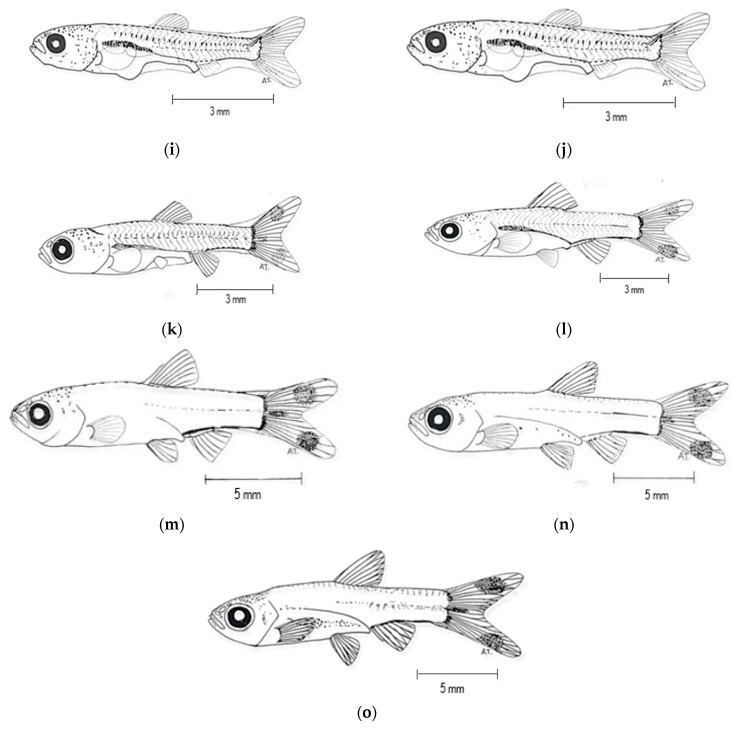
Larval development of scissortail rasbora (*Rasbora trilineata*): (**a**) Newly hatched larva, length 2.75 ± 0.35 mm; (**b**) 12 h old larva, length 3.43 ± 0.40 mm; (**c**) 1-day-old larva, length 3.84 ± 0.55 mm; (**d**) 2-day-old larva, length 4.52 ± 0.45 mm; (**e**) 3-day-old larva, length 5.00 ± 0.50 mm; (**f**) 5-day-old larva, length 5.53 ± 0.50 mm; (**g**) 7-day-old larva, length 6.13 ± 0.80 mm; (**h**) 9-day-old larva, length 7.20 ± 0.80 mm; (**i**) 12-day-old larva, length 7.85 ± 0.95 mm; (**j**) 15-day-old larva, length 9.48 ± 1.00 mm; (**k**) 19-day-old larva, length 10.43 ± 1.10 mm; (**l**) 24-day-old larva, length 12.45 ± 1.20 mm; (**m**) 29-day-old larva, length 17.00 ± 1.40 mm; (**n**) 35-day-old larva, length 18.30 ± 1.55 mm; (**o**) 40-day-old larva, length 20.00 ± 1.65 mm. **Note:** Length was measured as total length (TL).

Newly hatched *R. trilineata* larvae were straight, transparent, laterally compressed, and gradually tapered towards the tail. The common size at hatching was about 2.75 ± 0.35 mm in TL and could reach 6 ± 0.80 mm in TL within 7 days (Figure 4). One day after hatching (DAH), the size increased by about 1 mm and reached about 3.8 ± 0.55 mm in TL. Growth was linear from 1 to 40 DAH (Figure 5). The changes in the larval development of *R. trilineata* during the period from 1–40 DAH are shown in Figure 6. In the first 2 days, the *R. trilineata* larvae still possessed a yolk sac and were motionless near the water surface. The yolk sac was completely resorbed at 3 DAH, and the larvae developed fins and other structures, allowing them to swim independently and start feeding. Details of daily development are presented in Table 2.

## 4. Discussion

The present study demonstrated the successful induced breeding of *R. trilineata* in captivity using hormones, along with its development until it reached the adult stage at 40 DAH. *Rasora* spp. are reproductively non-seasonal, like many tropical cyprinids, but reproduction commonly peaks during the rainy season when environmental factors are suitable [25]. The wild fish samples in this study were collected during a period of intense rainfall in the study area. This fish species has no genital papillae for sex segregation. The differences in the sex of fish can be seen from their morphology and gonads. Female fish are relatively larger, while male fish have slender bodies. When massaged on the abdomen, the male fish will release white liquid sperm, while the female fish will release yellowish eggs [13]. It requires much experience to discriminate between male and female broodstock based on their body shapes. In our preliminary phase, many fish were sacrificed to examine their primary sex organs, i.e., gonads, to confirm the sex identification of individual specimens. Injecting fish requires considerable skill [17]. As a small and delicate species, mortality after injection can be high. It is better to gently handle the broodstock with a soaked towel and inject them while in a tray filled with water. The water should cover the fish to be injected by about 3–5 cm to reduce stress and hence lessen mortality (Figure S1).

Inducing maturation by hormone injection has been successful in many Rasborin cyprinids [15,16,26]. In the current study, dosages of Suprefact^®^ at 15 and 7.5 µg per kg body weight of *R. trilineata* for females and males, respectively, i.e., Treatment #3, revealed optimal results in terms of the number of spawned eggs and the fertilization rate. Shading was provided to replicate nature, as this fish spawns under dark conditions [5]. The results also confirm the efficacy of using a synthetic hormone to induce maturation of Rasborin cyprinids. The number of spawned eggs of *R. trilineata* in this study at the best dosage was about 6 times higher than the least effective treatment, Treatment #1. Ningrum et al. [26] reported success in using Ovaprim^tm^ to induce maturation of *Rasbora argyrotaenia*, which was injected at a dosage of 0.7 mL per kg for females. This resulted in a 1.5 times higher number of spawned eggs compared to the control, with no injected Ovaprim^tm^ [27]. It was revealed that injecting *Rasbora einthovenii* with synthesized GnRHa (at 15 µg per kg of fish) and a domperidone antagonist (at a 10 µg/kg dosage) for females resulted in a higher number of spawned eggs compared to the specimens that received no injection. Chlorella was among the best feeds during the post-embryonic period. This is because of its availability, and it is rich in essential fatty acids for fish larvae [28]. Moreover, it does not have any negative effects on growth, survival, or immune system function in fish [29]. The group fed with Moina had a higher larval survival from 14–40 DAH. Joshua et al. [30] reviewed the advantages of Moina compared to artificial feeds that induce jerking and whimsical movement, which makes it a noticeable prey. This is similar to the preferred feed of *R. trilineata* in the wild, which is exogenous insects [5]. Higher survival of the Moina-fed group should be because Moina contains more protein (ca. 70%) [6,30] compared to the other artificial feeds in this study, 40–50% protein, as presented in the Section 2.

For most cyprinids, the adhesiveness of the fertilized *R. trilineata* eggs is likely an adaptive trait facilitating substrate attachment in natural habitats. This enhances embryo survival by reducing drift and predation risk [31]. The developmental process of *R. trilineata* was completed within 18 hpf. The cleavage stage lasted for about 1.5 hpf, close to the time at this stage for *R. lateristriata* [18], *R. daniconius* [19], and *R. argyrotaenia* [20]. The rapid onset of cleavage indicates high metabolic activity and robust activation of the zygotes [24]. Subsequent stages, including the morula, blastula, and gastrula phases, revealed classic morphogenetic events such as epiboly and germ layer formation. The dome-shaped organization of cells at the blastula stage, along with the formation of the embryonic shield during gastrulation, occurs in other *Rasboras* and most cyprinids [32,33,34,35]. The segmentation period of *R. trilineata* was marked by the appearance of somites, along with head and tail buds. The shorter period of somite and myotome development in *R. trilineata* than other reported Rasborin cyprinids [18,19,20] suggests that these events are likely influenced by the ambient water temperature. The pharyngula stage in *R. trilineata* was observed at approximately 17 hpf, followed by hatching at 18 hpf. Completion of embryogenesis within 18 h places *R. trilineata* among the fast-developing freshwater teleosts, which normally require between 20–24 h [23]. This may confer ecological advantages such as reduced vulnerability to egg predation and environmental stressors [18,24,33].

Our findings show that the transition from endogenous to exogenous feeding and the subsequent development of morphological traits such as fin formation, pigmentation, and scale coverage follow a pattern comparable to other *Rasbora* spp. and other fishes in the Cyprinidae family [18,19,20,32,33,34]. The size of the newly hatched *R. trilineata* is similar to other Rasborin cyprinids, with reported initial lengths ranging from 2 to 3 mm in TL with limited mobility and incomplete organ systems [18,19,20]. Resorption of the yolk sac in *R. trilineata* was completed at 3 DAH, along with the appearance of a rudimentary mouth. Early feeding competency of fish larvae typically coincides with yolk depletion. This is crucial for ensuring larval survival and growth [36]. This finding is relevant for ornamental aquaculture practices, as this size coincides with the minimum threshold for handling and transport [37].

## 5. Conclusions

Artificial breeding of scissortail rasbora *Rasbora trilineata* was successful using the synthetic hormone Suprefact^®^. Feeding the newly hatched *R. trilineata* for 14 days is suggested, followed by Moina until 40 DAH, which resulted in a high survival rate. The development stages of *R. trilineata* are classified as zygote, cleavage, morula, blastula, gastrula, segmentation, pharyngula, and hatching. The larvae showed rapid linear growth, attaining 20 ± 1 mm in TL within 40 days. Future research should focus on optimizing rearing conditions and genetic diversity assessments to enhance the species’ commercial viability and conservation efforts.

## Figures and Tables

**Table 1:** Approximate time and explicit remarks at each stage of embryonic development of scissortail rasbora (*Rasbora trilineata*).

Embryonic Stages	Approximate Time *	Explicit Remarks During Development
Zygote	10 min	Cell division begins.
Cleavage		
• 2-cell	14 min	Division of 1 cell into 2 cells.
• Second cleavage	17 min	Division of 2 cells into 4 cells.
• Third cleavage	20 min	Division of 4 cells into 8 cells.
• Fourth cleavage	30 min	Continued cell division, entering the morula stage.
Morula	2 h, 30 min	Cells divide and arrange into multiple layers, resembling a mulberry-like structure.
Blastula	3 h, 30 min	Increase in the number of cells.
• Dome stage	4 h, 30 min	Cells arrange into a dome shape, transitioning towards a horizontal alignment.
Gastrula	5 h	Cells organize into a germ ring (germ layer formation).
Segmentation		
• Head bud stage	8 h	The head bud and tail bud become visible.
• Primordium	8 h, 30 min	The optic bud appears in the head region.
• Somite stage	9 h	Smites begin to form along the body axis.
	11 h, 30 min	Somite count increases.
	12 h, 55 min	Formation of myotomes and initial muscle movements.
	15 h, 30 min	Movement of the body begins with the embryo wriggling inside the egg.
	11 h, 30 min	Somite count increases.
Pharyngula	17 h	Formation of the chorion membrane.
	18 h	Hatchling emerges from the egg as a free-swimming larva.

* Approximate time after fertilization.

**Table 2:** Description of larval development of scissortail rasbora (*Rasbora trilineata*).

Days After Hatching (DAH)	Development Description
Early hatched	The mouth has not developed, the yolk sac is fully present, there is a fin fold, and the body axis is straight.
12 h AH	The larval length increased while the yolk sac became smaller and slimmer at early hatching. Rudimentary pectoral fins begin to develop on each side of the body.
1 DAH	Spines begin to appear at the anterior portion of the larval.
2 DAH	The body is attached to the horizontal spinal cord.
3 DAH	The indentation of the mouth appeared, forming a gap. The yolk sac became thinner and crenulated below the stomach.
5 DAH	The larvae began to show more swimming activities at the bottom and the edge of the aquarium.
7 DAH	The rudimentary caudal fins started to develop.
9 DAH	Melanophores were spotted at the operculum near the head of the larvae. The lateral line was visible from the head to the tail.
12 DAH	The operculum became thicker and fully covered the gills. Caudal and dorsal fins started to develop, and the caudal fin became forked in the middle.
15 DAH	The mouth had fully developed, and feeding began at 15 DAH with Moina. Melanophores were spotted at the caudal fin.
19 DAH	Pectoral fins, anal fins, dorsal fin, and caudal fin had fully developed.
24 DAH	Pelvic fins started to develop, and their size increased.
29 DAH	The fish reached the juvenile stage as early as 28–30 DAH. Scales fully covered its body. Fin shapes and colors were similar to adults.
35 DAH	The fish behavior was similar to that of adult *R. trilineata*, showing a uniform swimming pattern, swimming in schools.
40 DAH	The fish could be fed with artificial feed, and they had reached the minimum size for export.

## Data Availability

The data that support the findings of this study are available on request from the corresponding author.

## Supplementary Materials

The following supporting information can be downloaded at: https://www.mdpi.com/article/10.3390/ani15131823/s1, Table S1: Ranges of water quality parameters during the experiment period; Figure S1: Injection protocols for *Rasbora trilineata*.

## Abbreviations

The following abbreviations are used in this manuscript:

DAH	Day after hatching
hpf	Hours post fertilization
TL	Total length
