# Supplementary material for: Breeding and Ontogeny of the Aquarium-Traded Scissortail Rasbora (Rasbora trilineata)
Source: Animals (Basel). 2025 Jun 20;15(13):1823. doi: 10.3390/ani15131823 (PMC12248566; doi:10.3390/ani15131823)
Supplement: Supplementary file 1 [file animals-15-01823-s001.zip › animals-3661472-supplementary.pdf]

Table S1. Ranges of water quality parameters during the experimental period.

Parameter	Water quality
Temperature (c°)	24.5±0.37–28.0±0.35
Dissolved Oxygen (ppm)	4.5 ±0.21–6.0 ±0.15
pH	6.5 ±0.18–7.5 ±0.22
Alkalinity (ppm)	55 ±2.19–68 ±1.41
Hardness (ppm)	80 ±3.41–95 ±1.67
Nitrate; NO_6# (ppm)	0
Ammonia; NH_3 (ppm)	0

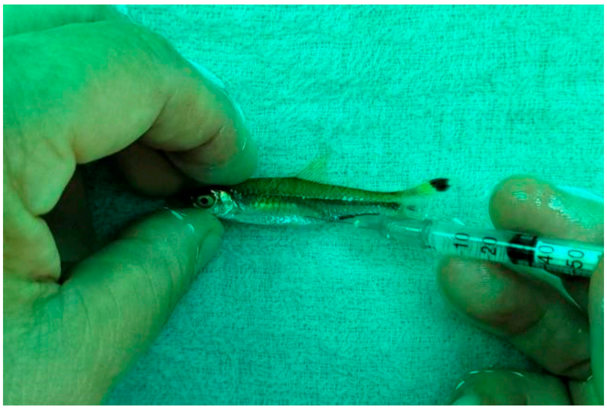

Figure S1. Injection performed on *Rasbora trilineata.*
